# Keep Politics out of Funding Decisions for Medical Research and Public Health

**DOI:** 10.4269/ajtmh.20-0850

**Published:** 2020-07-22

**Authors:** Philip J. Rosenthal, Daniel G. Bausch, Karen A. Goraleski, David R. Hill, Julie A. Jacobson, Chandy C. John, Joel G. Breman

**Affiliations:** 1 Department of Medicine, University of California San Francisco, San Francisco, California;; 2 United Kingdom Public Health Rapid Support Team, Public Health England and London School of Hygiene and Tropical Medicine, London, United Kingdom;; 3 American Society of Tropical Medicine and Hygiene, Arlington, Virginia;; 4 Frank H. Netter School of Medicine, Quinnipiac University, Hamden, Connecticut;; 5 Institute for Global Public Health, Quinnipiac University, Hamden, Connecticut;; 6 Bridges to Development, Seattle, Washington;; 7 Ryan White Center for Pediatric Infectious Diseases and Global Health, Indiana University School of Medicine, Indianapolis, Indiana

Authors are members of the American Society of Tropical Medicine and Hygiene’s Executive Committee

The *American Society of Tropical Medicine and Hygiene* is the largest international scientific organization of experts dedicated to reducing the worldwide burden of tropical infectious diseases and improving global health. As leaders of the society, we are compelled to speak up for the integrity of science, and specifically for research funding and public health decisions based on merit, with policies rooted in data. Recently, key decisions have lacked this basis, and science is under attack.

On April 24, 2020, the NIH abruptly canceled funding for an ongoing program studying coronaviruses that had been funded since 2014. This decision was highly unusual for several reasons. First, the NIH almost never cancels grants after funding is approved based on rigorous peer review, unless there is financial or scientific misconduct. Second, it is remarkable that, of all grants to cancel in the midst of the COVID-19 pandemic, it is a grant to study coronaviruses. Third, it has been substantiated that the decision to cancel the grant came not from the NIH but from the highest levels of the U.S. government. There is little doubt that a productive research program, indeed a program directly relevant to our attempts to control the greatest respiratory virus pandemic of the last century, was canceled because of political pressure. Canceling a major coronavirus research program at exactly the time when more, not less, research in this area is needed is deeply disturbing.

The research program that was canceled was led by the EcoHealth Alliance, a U.S.-based global nonprofit organization that conducts research on five continents in biosurveillance, deforestation, wildlife conservation, and, most relevant to this conversation, zoonotic diseases and pandemic prevention. This research, which included collaboration with other international institutions, focused on coronavirus spread from bats to other species, including humans. The research results contributed to studies to prevent the spread of and to develop drugs and vaccines against SARS-CoV-2, the cause of COVID-19.^1^ EcoHealth Alliance research has helped us to understand how coronaviruses jumped from bats to humans to cause the current pandemic; the canceling of this program without scientific basis seems a flagrant example of “shooting the messenger.”

The reason for cutting the coronavirus research project is quite transparent. The Trump administration reportedly learned about the project, including its links to the Wuhan Institute of Virology, from a reporter on April 17. Soon thereafter, it was announced that the program would be cut. The Department of Health and Human Services justified the cut with the claim that “the grantee was not in compliance with NIH’s grant policy,” but the director of the NIH Institute that funded the study later stated in congressional testimony that “it was canceled because the NIH was told to cancel it.” Clearly, the actual reason for cancellation was political, apparently based on an attempt to assign blame for the COVID-19 pandemic to China, and consistent with claims that the pandemic was the result of a laboratory accident or a deliberate attempt to initiate a viral outbreak in Wuhan. These claims are unsubstantiated and readily refuted by available molecular data.^1^

On another front, on May 29, the White House announced that the United States will terminate its relationship with the WHO; this was confirmed on July 6 when the United States sent an official withdrawal letter to the United Nations. The WHO was founded in 1948 to coordinate international health policy, establish global disease surveillance networks, and lead disease control and eradication programs. It is supported by and under the control of its 194 member states. Although burdened through its history by limited control over international health policy and bureaucratic constraints, overall, the WHO has been remarkably successful and is an essential global institution. The WHO played a pivotal role in the eradication of smallpox, it has led successful efforts to control and eliminate many other infectious diseases, and it is the key international agency to lead efforts to address international outbreak and epidemic diseases, from influenza to HIV/AIDS, multidrug-resistant tuberculosis, polio, onchocerciasis, guinea worm, Ebola, Zika, and now COVID-19.

The reason for cutting ties with the WHO seems as clear as that for the NIH grant cut described previously. With a failing response to the COVID-19 pandemic at home and a pending election, the U.S. administration acted to deflect criticism to the WHO. Although initial international responses to the emerging pandemic may have been slower than optimal, the WHO played a major role in rapid dissemination of information regarding virus isolation and characterization within a few weeks of recognition of the outbreak, oversaw the rapid development of reliable tests for the infection, and has regularly written and updated evidence-based guidance for control of the pandemic. WHO coordination and strong national programs and leadership are helping to curb COVID-19 in many countries. As with cutting funding for coronavirus research during the pandemic, cutting funding for the WHO is profoundly troubling.

At this time of deep human tragedy and economic disaster due to the COVID-19 pandemic and of political upheaval in many countries, the headlines change quickly, and politically motivated mandates of great importance can fail to generate the attention that they deserve. We have therefore highlighted two recent decisions by the White House that are profoundly misguided. Indeed, they seem the worst possible choices as we grapple with an overwhelming pandemic. But these choices can be reversed. We urge President Trump to 1) reinstate and work with Congress to increase funding for essential research on coronaviruses to help us develop new tools and strategies to address current and future pandemics, and 2) continue the country’s long-standing relationship with the WHO, offering full financial support and cooperation for this essential international body. More broadly, we implore our elected officials at every level of government to keep politics out of decisions regarding medical research and public health. The health of Americans and of the population of our planet must not be a bargaining chip used to seek political gains or deflect accountability. Rather, health is an indisputable human right. As we fight the worst respiratory pandemic of the last century, it is foolhardy to limit support for the best research and the best public health institutions that are working to stem the pandemic.
